# Adrenocortical carcinosarcoma: a case report and review of the literature

**DOI:** 10.1186/1746-1596-5-51

**Published:** 2010-08-05

**Authors:** Kotaro Sasaki, Marisa Desimone, Harsha R Rao, George J Huang, Raja R Seethala

**Affiliations:** 1 Department of Pathology, University of Pittsburgh Medical Center, Pittsburgh, PA, 15232 USA; 2 Department of Medicine, Division of Endocrinology and Metabolism, University of Pittsburgh Medical Center, Pittsburgh, PA 15232 USA; 3 Department of Urology, University of Pittsburgh Medical Center, Pittsburgh PA 15232, USA

## Abstract

Adrenocortical carcinosarcoma is an extremely rare and aggressive variant of adrenocortical carcinoma characterized by the presence of both carcinomatous and sarcomatous components, with the latter often showing heterologous differentiation. Due to the rarity and unusual histology, it may pose a diagnostic challenge. In order to increase awareness and identify potential diagnostic pitfalls, we report the ninth case of non-functioning adrenocortical carcinosarcoma in a 45-year-old man who presented with worsening epigastric pain and a left large retroperitoneal mass in close proximity to the body/tail of pancreas and third portion of the duodenum with displacement of the kidney without parenchymal invasion and multiple liver nodules detected by computed tomographic scan. On en bloc resection, the tumor grossly did not involve the pancreas, kidney or colon. Histologically, the tumor was composed of two distinct components - an epithelioid component with granular cytoplasm that stained for synaptophysin, Melan-A, calretinin, and vimentin compatible with adrenocortical differentiation, and a pleomorphic to spindled component that was positive for desmin and myogenin, compatible with rhabdomyosarcomatous differentiation. A wedge biopsy of a liver nodule showed morphologic features similar to the epithelial component of the primary tumor. The patient died three months after surgery due to locoregional and distant recurrence. Adrenocortical carcinosarcoma is a rare malignancy that adds to the differential diagnostic considerations for a retroperitoneal epithelioid malignancy. Awareness of this as a possibility will help in distinguishing this tumor from other carcinomas, melanomas, and true sarcomas.

## Background

Adrenocortical carcinoma is a rare but highly aggressive malignancy with an estimated annual incidence of between 1.5 to 2 per million population [1]. Women are more commonly affected. There is a bimodal age distribution with cases a peak occurring before age 5 years and a second in the fourth to fifth decades [2]. The prognosis is poor with a significant proportion (21% to 39%) of patient having distant metastasis at the time of presentation [2,3] and a 5 year overall survival ranges between 38% to 60% [1]. Even after an apparently curative resection, the majority of patients develop early tumor recurrence or distant metastasis [1-3].

Carcinosarcomas are defined as malignant neoplasms showing both epithelial and mesenchymal differentiation with heterologous features including rhabdomyoblastic, chondroid, or osteogenic differentiation [4]. We report a case of primary adrenal carcinosarcoma and review the literature to raise awareness of this extremely rare variant of adrenal carcinoma with worse prognosis presenting high differential diagnosis difficulties.

## Case Presentation

### Case History

**Figure 1:**
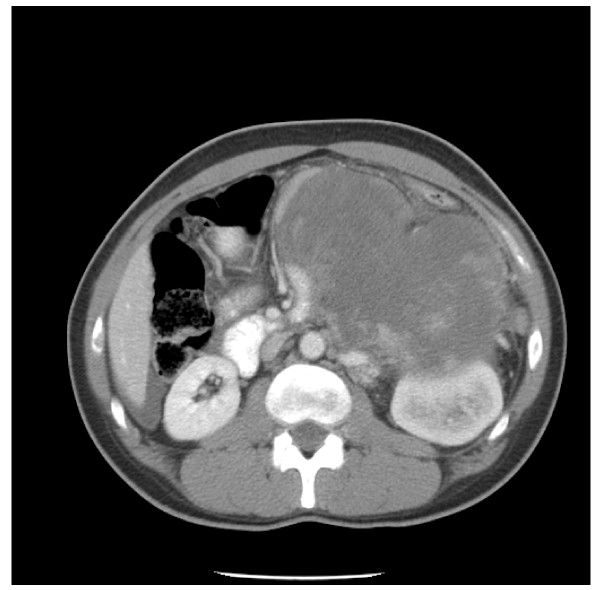
**CT scan of adrenocortical carcinosarcoma**. Necrotic left adrenal mass in close proximity to the body/tail of pancreas and third portion of the duodenum with displacement of the kidney

A 45 year-old African American male with no past medical history of hypertension or prior malignancy was admitted to the hospital with worsening epigastric pain, low-grade fever, nausea, vomiting, decreased appetite, and 9 kg loss of weight over three months. On physical examination, his blood pressure was 189/119 mm Hg. His abdomen was mildly distended. A dynamic, contrast-enhanced abdominal computed tomography scan revealed a large (19 × 15 cm) irregularly and peripherally enhancing predominantly necrotic left retroperitoneal mass in close proximity to the body/tail of pancreas and third portion of the duodenum with displacement of the kidney without parenchymal invasion (Fig 1). Extensive bilobar hepatic metastatic lesions were also noted but no lymphadenopathy was seen. The laboratory studies did not show significant steroid hormone or catecholamine excess. No other mass lesions were noted in the patient.

An en-bloc resection of the mass which included a left radical nephrectomy, splenectomy, distal pancreatectomy, left partial colectomy, and wedge biopsy of one of the hepatic lesions were performed. However, despite this, at 3 months, the patient had a locoregional recurrence and progression of liver disease. Due to his poor performance status (Eastern Cooperative Oncology Group performance status 3), no chemotherapy was performed. The patient died 3 months after the surgery. Autopsy was not performed.

### Gross Examination

**Figure 2:**
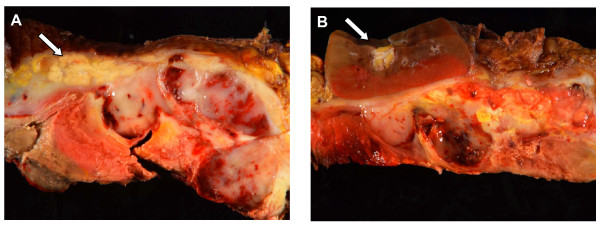
**Adrenocortical carcinosarcoma gross appearance**. A. Tumor adherent to pancreas (arrow) without apparent parenchymal involvement. B. Tumor compressing the adjacent left kidney (arrow) without parenchymal involvement.

The gross specimen consisted of a centrally necrotic, peripherally viable appearing, heterogenous gray to pink-yellow friable suprarenal mass, 17.0 × 6.0 × 6.0 cm, 2974 grams, completely effacing the adrenal gland (Fig. 2A). Pancreas and kidney were adherent to tumor but otherwise uninvolved by tumor (Fig 2B). Spleen and colon were uninvolved by tumor.

### Microscopic Examination

**Figure 3:**
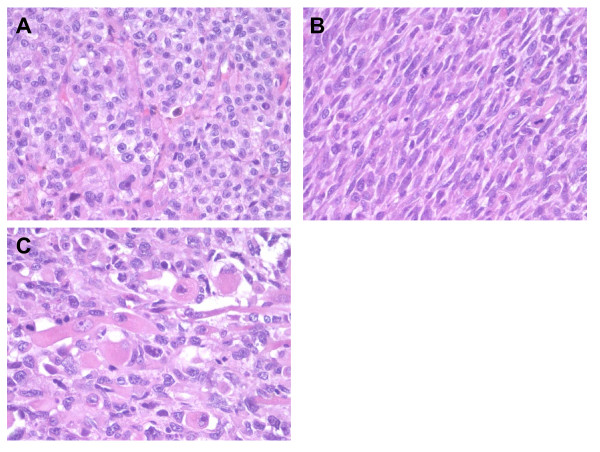
**Adrenocortical carcinosarcoma histological appearance**. A. Carcinomatous component showing epithelioid cells with clear to eosinophilic cytoplasm arranged in nested pattern (40×). B. Sarcomatous component composed of uniform spindle cells arranged in a fascicular pattern (40×). C. Sarcomatous component showing elongated or ovoid rhabdomyoblastic tumor cells with eccentrically located nuclei and deeply eosinophilic cytoplasm (20×).

The tumor showed extensive necrosis (over 70%). Two components were noted, an epithelioid component, and a pleomorphic/spindled component. The epithelioid component consisted of sheet and nests of loosely cohesive polygonal cells with clear and eosinophilic cytoplasm resembling adrenocortical cells (Fig. 3A). These cells showed highly atypical nuclei and large eosinophilic nucleoli (Fuhrman's grade III) with high mitotic activity (average of seven mitoses per 10 high-power fields). The pleomorphic/spindled component, comprising approximately 75% of viable tumor, showed predominantly spindle-shaped cells arranged in fascicular pattern with highly pleomorphic nuclei with dense irregularly clumped chromatin and prominent dense eosinophilic nucleoli (Fig. 3B) and occasional anaplastic multinucleated neoplastic giant cells. Also identified in these areas were large elongated or ovoid cells with abundant deeply eosinophilic cytoplasm and eccentrically located nuclei and prominent nucleoli, suggestive of rhabdomyosarcomatous differentiation (Fig. 3C). The mitotic count in these areas was even higher (30 mitoses/10 high-power fields).

Histologically as well, there was no involvement of the pancreas, kidney, or colon, and the surrounding adipose tissue was unremarkable. The liver wedge biopsy showed a tumor nodule morphologically identical to the epithelioid component of the retroperitoneal tumor.

### Immunohistochemistry

**Figure 4:**
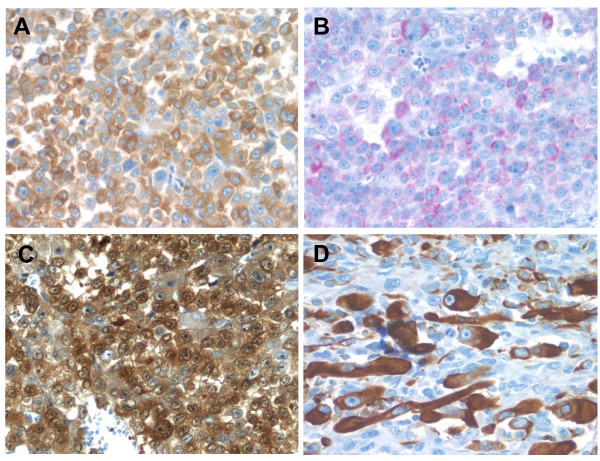
**Adrenocortical carcinosarcoma immunohistochemistry**. A. The carcinomatous areas are strongly positive for synaptophysin (40×). B. Melan-A shows patchy positivity in the carcinomatous areas (40×). C. Calretinin immunostain showing diffuse cytoplasmic and nuclear positivity in carcinomatous areas (40×). D. Desmin immunostaining highlighting rhabomyoblastic cells in sarcomatous area (40×).

An extensive immunohistochemical panel was performed to evaluate both epithelioid and pleomorphic spindled components of tumor (Table 1). The epithelioid component was strongly and diffusely positive for vimentin, synaptophysin (Fig. 4A), and Melan-A (Fig. 4B) and focally positive for calretinin (Fig. 4C). The pleomorphic/spindled component was strongly and diffusely positive for vimentin, focally strongly positive for synaptophysin, Melan-A, and focally weakly positive for calretinin. The cells resembling rhabdomyoblasts were immunoreactive for smooth muscle markers, desmin (Fig. 4D), myogenin, and myoglobin. Immunohistochemical stains for AE1/AE3, S-100, HMB-45, tyrosinase, inhibin, PLAP, CD99, and chromogranin were negative in both carcinomatous and sarcomatous components.

**Table 1:** Adrenocortical carcinosarcoma immunohistochemical profile

Stain	Carcinomatous component	Sarcomatous component	Company	Clone	Dilution
**AE1/AE3**	Negative	Negative	Dako	AE1/AE3	1:100

**Vimentin**	Positive	Positive	Ventana	V9	Prediluted

**Desmin**	Negative	Positive	Ventana	DE-R-11	Prediluted

**Myogenin**	Negative	Weakly Positive	Cell Marque	F5D	Prediluted

**Myoglobin**	Negative	Positive	Ventana	Polyclonal	Prediluted

**S-100**	Negative	Negative	Dako	Polyclonal	1:500

**HMB-45**	Negative	Negative	Ventana	HMB45	Prediluted

**Tyrosinase**	Negative	Negative	Vector	T311	1:75

**Melan-A**	Focally weakly positive	focally positive	Dako	A103	1:100

**Synaptophysin**	Positive	focally positive	Dako	Cell Marque	Prediluted

**Chromogranin**	Negative	Negative	Ventana	LIK2H10	Prediluted

**Inhibin Alpha**	Negative	Negative	Dako	R1	1:50

**PLAP**	Negative	Negative	Biogenex	PL8/F6	1:400

**CD99**	Negative	Negative	Dako	12E7	1:75

**Calretinin**	Positive	Focally positive	Invitrogen	Polyclonal	1:100

Based on the histologic and immunohistochemical profile, the tumor was diagnosed as an adrenocortical carcinosarcoma.

## Discussion

Adrenocortical carcinoma containing a component of sarcoma or sarcoma-like (spindle cell) differentiation is extremely rare, with only 8 prior cases described [5-12]. According to WHO classification 2004 in other epithelial malignant neoplasms, these tumors are classified as sarcomatoid carcinoma. Within the broad category of sarcomatoid carcinoma, tumors with histological areas of both carcinoma and sarcoma containing differentiated sarcomatous elements, such as malignant cartilage, bone or skeletal muscle are subclassified as carcinosarcoma. To our knowledge, only 3 prior cases of carcinosarcoma have been reported [7-9].

The clinicopathologic findings of all adrenocortical sarcomatoid carcinomas, including our patient, are summarized in Table 2. The 9 patients ranged from ages 29 to 79 with a mean age of 53, which appears similar to conventional adrenocortical carcinoma (mean age 40-50 years old). Although most studies of conventional adrenocortical carcinoma have a female preponderance [2], sarcomatoid carcinoma show nearly equal distribution of two sexes. Initial clinical presentation is most often flank/abdominal pain or discomfort (six of 9 cases). Tumors tend to be very large at the time of initial presentation (mean size 13.0 cm, weight 1113 grams). Two of 9 (22%) tumors were functional with production of dehydroepiandrosterone sulfate or aldosterone. All nine cases were treated with surgical resection often combined with systemic adjuvant chemotherapy, however, all patients died within 1 year (mean postoperative survival 5 months). Including our case, only four of 9 tumors (44%) were actually carcinosarcomas most often with rhabdomyosarcoma (3/4) followed by mixture of osteosarcoma and chondrosarcoma (1/4). Though difficult to say based on small numbers, adrenocortical carcinosarcomas and other sarcomatoid carcinomas without heterologous elements show similar distribution of age (mean age 48.7 versus 56.2), tumor size (13.3 cm vs. 12.8 cm), and outcome (mean survival: 6 months vs. 5.4 months).

**Table 2:** Clinicopathologic features of adrenocortical sarcomatoid carcinoma

Author	Age	Sex	Clinical Presentation	Endocrine dysfunction	Size (cm) weight (g)	Sarcomatous component	postoperative time of death
Okazumi et al. (1987)	46	M	Abdominal distention	No	14 cm, 880 g	Spindle	6 months

Collina et al. (1989)	68	F	Abdominal discomfort	No	11 cm	Spindle	6 months

Decorato et al. (1990)	42	F	Abdominal pain	No	19 cm, 1400 g	Rhabdomyosarcoma	7 months

Fischer et al. (1992)	29	F	Virilization, weight loss	Yes	12.5 cm, 610 g	Rhabdomyosarcoma	8 months

Barksdale et al. (1993)	79	F	hypertension	Yes	5 cm, 199 g	Osteosarcoma, chondrosarcoma	NA

Lee et al. (1997)	61	M	Flank pain	No	12 cm	Spindle	2 days

Sturm et al. (2008)	31	M	Abdominal pain	No	12 cm, 620 g	Spindle	3 months

Coli et al. (2010)	75	F	Abdominal pain	No	15 cm	Spindle	12 months

This study	45	M	Abdominal pain	No	17 cm, 2974 g	rhabdomyosarcoma	3 months

Radiographically and even grossly, it is often difficult to confirm adrenal origin for these tumors due to the advanced presentation. Indeed, in two of 9 cases of sarcomatoid carcinoma, imaging studies could not correctly locate the adrenal origin of tumor. As such, the differential diagnostic considerations include other aggressive retroperitoneal malignancies including other carcinomas, particularly renal cell carcinoma, true sarcoma, large cell lymphoma, and metastases. Although some adrenocortical neoplasms produce steroid hormones, others are non-functional, which makes it difficult to identify specific adrenocortical tumor markers. In our case, hormone levels were unremarkable. Here, a thorough clinical history and precise characterization of structures involved may be useful in narrowing possibilities. In our case for instance, the pancreas, and kidney on thorough examination were grossly uninvolved arguing against these as primary sites of origin. There was no lymphadenopathy arguing against lymphoma, and there was no history of a prior malignancy or any other masses arguing against a metastasis.

The diagnosis of adrenocortical carcinosarcoma on histologic examination is often challenging as well. It requires thorough sampling of the specimen to confirm the biphasic pattern and identify a well differentiated carcinomatous component allowing to prove the adrenal origin as well as to rule out retroperitoneal sarcoma or poorly differentiated carcinoma.

The adrenal phenotype of this tumor was verified by the immunopositivity for a panel of immunohistochemical markers, namely, Melan-A [13], synaptophysin [14], calretinin [15], particularly on the well differentiated carcinomatous component. Of note, in contrast to most carcinomas, adrenocortical carcinomas are notoriously negative or only focally weakly positive for cytokeratins. In this study, we showed that sarcomatous component of the tumor also focally retains positivity for Melan-A, synaptophysin and calretinin, supporting the notion that sarcomatous area of the tumor has indeed originated from the adrenocortical carcinoma rather than representing a collision tumor. The sarcomatous component seen in our case contains frequent foci of rhabdomyoblastic cells. These foci could be sharply highlighted by desmin, myogenin and myoglobin, which is both a sensitive and specific marker for myogenic differentiation [16].

Similar to the clinicoradiographic diagnostic considerations, the histologic differential diagnostic considerations include 3 basic categories - other carcinosarcomas from other sites, most notably sarcomatoid renal cell carcinoma, true primary retroperitoneal soft tissue tumor, and metastases with sarcomatoid elements such as a germ cell tumor or rarely melanoma. Sarcomatoid renal cell carcinoma or hepatocellular carcinoma with sarcomatoid dedifferentiation both may show morphologically similar appearance with clear and eosinophilic cytoplasm. However, positive staining of CD56, inhibin, Melan-A, synaptophysin, calretinin and negative staining of pan-cytokeratin, EMA, Hepar-1 in adrenocortical sarcomatoid carcinoma may be of help in the differential diagnosis [17,18]. A primary retroperitoneal sarcoma such as liposarcoma, rhabdomyosarcoma, or malignant peripheral nerve sheet tumor also needs to be excluded by careful histological and immunohistochemical analysis. The lack of well-differentiated liposarcomatous component and presence of well-differentiated adrenocortical carcinoma component excludes the possibility of de-differentiated liposarcoma. In difficult cases, immunohistochemical, fluorescence in situ hybridization or quantitative PCR analysis for CDK4 and MDM2 status may be of interest [19]. Rhabdomyosarcoma is usually a neoplasm of children/infants and lacks well-differentiated adrenocortical carcinoma component. Malignant peripheral nerve sheet tumor with rhabdomyoblastic elements (Triton tumor) can be excluded by morphology as well as negativity of Melan-A, synaptophysin, and calretinin and positivity of S-100 [20,21].

Metastatic melanoma with heterologous elements might enter the differential diagnosis since this will also be positive for Melan-A and negative for cytokeratin. This can be distinguished from an adrenocortical tumor by positivity for other melanocytic makers such as S-100, HMB-45, tyrosinase and negativity of calretinin, synaptophysin and inhibin [22]. It is of note, however, that Coli et al. reported unusual positive staining of S-100 and HMB-45 in adrenocortical sarcomatoid carcinoma, which has not been reported previously for adrenocortical tumors. Although they interpreted this immunohistochemical pattern as aberrant melanocytic differentiation, further studies may be needed to confirm this unusual expression of S-100 and HMB-45. Mixed germ cell tumor, most often metastasis from testicular primary, with somatic teratomatous malignancy (rhabdomyosarcoma) can be excluded by positive staining of PLAP and cytokeratins and negative staining of vimentin, Melan-A and calretinin [23].

Together, based on our literature review, carcinomatous component of adrenocortical sarcomatoid carcinoma is immunoreactive for Melan-A (2 of 3 cases, 67%), synaptophysin (2 of 4 cases, 50%), calretinin (2 of 4 cases, 50%), inhibin (1 of 3 cases, 33%), vimentin (5 of 5 cases, 100%), Neuron-specific enolase (NSE) (2 of 2 cases, 100%), occasionally positive for AE1/AE3 (1 of 5 cases, 20%), and negative for chromogranin (0 of 3 cases, 0%), EMA (0 of 2 cases, 0%), and neurofilament (0 of 2 cases, 0%). Sarcomatous component is positive for desmin (4 of 4 cases, 100%), myogenin (2 of 2 cases, 100%), HHF35 (2 of 2 cases, 100%), vimentin (6 of 6 cases, 100%), myoglobin (1 of 1 case, 100%), caldesmon (1 of 1 case, 100%), smooth muscle actin (1 of 2 cases, 50%), calretinin (1 of 2 cases, 50%), Melan-A (1 of 3 cases, 33%), occasionally synaptophysin (1 of 4 cases, 25%) and negative for AE1/AE3 (0 of 5 cases, 0%), EMA (0 of 2 cases, 0%), HMB-45 (0 of 2 cases, 0%), inhibin (0 of 3 cases, 0%), chromogranin (0 of 3 cases, 0%).

## Conclusion

In conclusion, we have reported the ninth case of adrenocortical sarcomatoid carcinoma with rhabdomyoblastic differentiation (carcinosarcoma). This lesion is often difficult to distinguish from other retroperitoneal neoplasms by radiographic imaging and is a highly aggressive form of adrenocortical malignancy. Thorough sampling, careful histological examination and widely extensive immunohistochemical investigation are often necessary to confirm adrenocortical origin and distinguish this tumor from other retroperitoneal sarcomatoid neoplasms.

## Consent

Written informed consent was obtained from the patient for publication of this case report and accompanying images. A copy of the written consent is available for review by the Editor-in-Chief of this journal.

## Competing interests

The authors declare that they have no competing interests.

## Authors' contributions

KS designed the study, performed the histopathological evaluation, literature review, acquired photomicrographs, and drafted the manuscript. MD, HRR and GJH participated in analysis and interpretation of data. RRS conceived and designed the study, gave and reviewed the final histopathological diagnosis, and revised the manuscript for important intellectual content. All authors read and approved the final manuscript.
